# Chinese herbal medicine for impaired glucose tolerance: a randomized placebo controlled trial

**DOI:** 10.1186/1472-6882-13-104

**Published:** 2013-05-14

**Authors:** Suzanne J Grant, Dennis Hsu-Tung Chang, Jianxun Liu, Vincent Wong, Hosen Kiat, Alan Bensoussan

**Affiliations:** 1 Centre for Complementary Medicine Research, School of Science and Health, University of Western Sydney, Locked Bag 1797, Penrith, NSW 2751, Australia; 2 Research Centre, Xiyuan Hospital, No 1 Xiyuan Cao Chang, Haidian District, Beijing, China; 3 Department of Endocrinology, Liverpool Hospital, South Western Sydney Area Health Service, Sydney, Australia; 4 The Australian School of Advanced Medicine, 2 Technology Place, Macquarie University, North Ryde, NSW 2109, Australia

## Abstract

**Background:**

Diabetes remains a major health problem worldwide. Low-risk low-cost alternatives to pharmaceutical interventions are needed where lifestyle modifications have failed. We conducted a double-blind randomised placebo controlled trial to investigate the efficacy of a Chinese herbal formula, *Jiangtang Xiaozhi*, in treating impaired glucose control and insulin resistance in persons with prediabetes and controlled diabetes.

**Methods:**

Seventy-one patients with prediabetes or ‘controlled’ diabetes were randomised to receive 3 capsules of *Jiangtang Xiaozhi* (n = 39) or placebo (n = 32) three times daily for 16 weeks with a follow up eight weeks later (week 24). The primary outcome was change in glycaemic control as evidenced by fasting blood glucose (FBG), post-prandial plasma glucose and glycosylated haemoglobin (HbA1c). Other measures included change in fasting insulin, insulin resistance and sensitivity, lipids, C-reactive protein (CRP), body mass index (BMI), waist girth, blood pressure (BP), health related quality of life (HRQoL) and safety. Analysis of covariance (ANCOVA) was used to model outcomes at 16 weeks, by treatment group corrected for baseline level of the outcome variable.

**Results:**

In patients receiving *Jiangtang Xiaozhi*, FBG was not significantly different (p = 0.73) compared to placebo after 16 weeks of treatment (6.3 ± 1.1 mmol/L vs 6.7 ± 1.3 mmol/L). There was a significant difference (p = 0.04) in the mean levels of fasting insulin between the treatment group (11.6 ± 5.5 mmol/L) and the placebo group (22.1 ± 25.9 mmol/L). Insulin resistance slightly decreased in the treatment group (1.58 ± 0.74) compared to that of the placebo group (2.43 ± 1.59) but this change did not reach statistical significance (p = 0.06). Patients taking *Jiangtang Xiaozhi* had a significant improvement in high-density lipoprotein (HDL) level compared to the placebo group at week 16 (p = 0.03). Mean levels of cholesterol, triglycerides, BMI, waist-girth, HRQoL, BP, CRP and insulin sensitivity were not significantly different between the two groups. The herbal medicine was well tolerated.

**Conclusions:**

In the current study, the 16 week *Jiangtang Xiaozhi* treatment did not lower fasting blood glucose, but it improved serum insulin and HDL cholesterol in a Western population with prediabetes or controlled diabetes. Our trial may have been underpowered. Dosage needs to be considered before commencing a longer adequately powered trial.

**Trial registration:**

Australian New Zealand Clinical Trials Registry ACTRN12612000128897; https://www.anzctr.org.au/Trial/Registration/TrialReview.aspx?id=362005

## Background

Worldwide it is estimated 285 million adults - equivalent to 6.4% of the population aged 20 to 79 yrs - have diabetes [1]. A further 344 million have impaired glucose tolerance (IGT) [1]. Over time, the glucose tolerance of many of these individuals will deteriorate and they will be diagnosed with diabetes. Impaired glucose tolerance, independent of diabetes, carries an increased risk of cardiovascular disease and all-cause mortality [2-4]. At present, the best course of action to reduce higher than normal blood sugar is to modify diet and increase physical activity. For some, rigorous and sustained behavioural change isn’t enough. In these cases pharmaceutical interventions such as metformin may be needed to delay or suppress the onset of diabetes. But this is not always an adequate long-term solution. A Kaplan-Meier analysis showed a cumulative incidence of monotherapy failure at 5 years of 15% with rosiglitazone, 21% with metformin and 34% with glyburide [5]. Low-risk low-cost alternatives to pharmaceutical interventions are clearly needed where lifestyle modifications have failed to adequately improve glucose tolerance.

Individuals with diabetes are 1.6 times more likely to use complementary and alternative medicine (CAM) than individuals without diabetes [6]. However these CAM interventions need to be tested in clinical trials to demonstrate efficacy and safety. Chinese herbal medicines have long been used for the treatment of IGT and diabetes in China, Korea and Japan, with anecdotal evidence of their effectiveness. In a meta-analysis of eight trials, those receiving Chinese herbal medicines with lifestyle modification were more than twice as likely to have their fasting plasma glucose levels return to normal compared to those receiving lifestyle modification alone [7]. Those receiving Chinese herbs were less likely to progress to diabetes over the duration of the trials. These trials were at considerable risk of bias due problems with randomisation, allocation concealment or blinding. Nonetheless the strength of the findings warrants further investigation.

*Jiangtang Xiaozhi* is a Chinese herbal formulation based on traditional Chinese medicine principles, modern research and clinical experience. Animal studies and a small clinical trial of *Jiangtang Xiaozhi*, along with studies of the effects of the individual herbs, have produced encouraging results [8-10]. In this article we report the findings from a randomized controlled trial evaluating the effect of *Jiangtang Xiaozhi* on blood glucose, insulin and lipids in people with IGT and controlled diabetes.

## Methods

### Patient and recruitment

Individuals were recruited across Sydney and the Central Coast of NSW, Australia through media (radio, television, newspapers), by approaching general practitioners, direct mail to specialised databases and presentations at forums for practitioners working in the field of diabetes. We included men and women over the age of 18 years of age with prediabetes or ‘controlled’ type 2 diabetes. Prediabetes is defined as having a fasting plasma glucose (FPG) level of <7.0 and 2 hr plasma glucose load level ≥ 7.8 and <11.0). ‘Controlled’ diabetes is not a standard medical diagnosis and was defined for the purpose of this study as people diagnosed within the last five years, whose diabetes was diet and exercise controlled and were not on any medication to control their blood glucose levels. Selection criteria were designed to ensure a heterogeneous population. We excluded individuals with conditions or treatments that would interfere with participation or completion of the protocol such as an underlying disease likely to limit life span or increase the risk of the intervention, or that had a confounding effect on the outcomes of the study, such as medication or a disease related to metabolism such as Cushing's syndrome. Baseline characteristics on age, sex, ethnicity, family history of diabetes, history of hypertension, smoking or use of cholesterol lowering or anti-hypertensive medication were collected at enrolment using an interviewer administered questionnaire.

Recruitment took place from June 2007 to December 2009. A formidable recruitment challenge was that prediabetes is asymptomatic and not recognised as a potentially serious condition. The trial was approved by the Human Research Ethics Committee at the University of Western Sydney, Australia. All participants gave written informed consent. This trial is registered with the Australian and New Zealand Clinical Trials Registry.

### Randomisation

A computer-generated randomisation list was used for treatment allocation in a 1:1 ratio. Randomisation was conducted by the UWS trial coordinator who was external to the trial. Participants and investigators were masked to group assignment. Unequal group size may have arisen due to one treatment occurring with greater frequency at the beginning of the randomisation list, as the study was terminated midway through the list [8]. The medication was sealed in sequentially numbered identical packets according to the allocation sequence. The UWS trial coordinator supplied labelled packets of the interventions as required. Participants and investigators were blind to the treatment allocated until the completion of data analysis.

### Herbal intervention and treatment schedules

*Jiangtang Xiaozhi* is comprised of six commonly used herbs. The herbs and dosage are shown in Table 1. The placebo and intervention were identical in appearance, taste and smell.

**Table 1:** **Composition of *Jiangtang Xiaozhi *capsules**

**Ingredient**	**Individual tablet**	**Dosage of 3 tablets**	**%**
Nu Zhen Zi (*Ligustrum lucidum Ait.;* Oleaceae; privet fruit)	1.33	4.00	35%
Huang Qi *(Astragalus membranaceus (Fisch.) BGE;* Fabaceae; milk vetch root)	0.67	2.00	18%
Huang Lian (*Coptis chinensis Franch.;* Ranunculaceae; coptis rhizome)	0.33	1.00	9%
Li Zhi He *(Litchi chinensis* SONN.; Sapindaceae; lyechee nut)	0.67	2.00	18%
Kun Bu *(Ecklonia kurome* OKAM.; Alariaceae; kelp*)*	0.06	0.17	1%
Jiang Huang *(Curcuma longa L.;* Zingiberaceae; tumeric rhizome*)*	0.50	1.50	13%
Lactose	0.02	0.05	1%
Magnesium stearate	0.21	0.64	6%
Total	3.79 g	11.36 g	100%

Both the *Jiangtang Xiaozhi* Capsule and the placebo were manufactured in China by Tianjin Zhongxin Pharmaceutical Group Corporation Ltd, a pharmaceutical manufacturer in China with an Australian Good Manufacturing Practice (GMP) license issued by the Therapeutic Goods Administration (TGA).

Participants were randomly allocated to receive either 3 *Jiangtang Xiaozhi* capsules or a placebo three times a day for 16 weeks. Participants were asked not to alter their diet or exercise habits during the intervention period. This was monitored at monthly visits. A double blind follow up visit was conducted 8 weeks after the completion of the treatment.

### Outcome measures

Primary outcomes were the change in fasting blood glucose (FBG), post-prandial plasma glucose and glycosylated haemoglobin (HbA1c) in the Chinese herbal medicine group from baseline to the conclusion of the trial compared to a placebo. Prediabetes is currently detected using FBG and followed up with a 2 hr oral glucose tolerance test (OGTT) to exclude diabetes [11]. The utility of HbA1c as a measure to detect and monitor prediabetes is currently being investigated and may supersede the combination of FBG and the OGTT [12]. All three tests (FBG, 2 hr OGTT and HbA1c) are highly correlated, therefore the inflation of the experiment-wise error rate arising from multiple testing will be slight [13]. The secondary outcomes were selected with a view to helping explain the primary outcome results, and shedding light on how the intervention might affect other risk factors for diabetes. The secondary outcomes were insulin, CRP protein, BMI and waist girth, lipids, blood pressure and health-related quality of life.

Fasting blood glucose, insulin and CRP were collected at 4 weekly intervals during the intervention. Post prandial glucose as measured by an OGTT was collected at baseline, trial completion (week 16) and at follow up (week 24). Fasting blood glucose and the OGTT was conducted after an overnight fast of at least 10–12 hours and 3 days of carbohydrate loading using a standard 75-g oral glucose tolerance test. HbA1c and lipids were measured at baseline, weeks 8, 16 and 24. Blood pressure, weight and waist girth were collected at weeks 0, 4, 8, 12, 16 and 24. Data on health related quality of life (HRQoL) was collected using the 36-item short-form health survey Version 2 (SF-36v2) at weeks 0, 16 and week 24 [14].

The Homeostatic model assessment (HOMA) was used to assess beta-cell dysfunction and insulin resistance [15]. HOMA%S is a measure of insulin sensitivity, HOMA%B is a measure of beta-cell function, and HOMA-IR is a measure of insulin resistance. The results of all three measures need to be reported together for proper interpretation. HOMA was selected as the model to use in this trial for two reasons. Firstly, it has been widely used and validated in a number of studies and has been found to correlate well with the euglycaemic clamp method [16]. Secondly, the sampling is simple, inexpensive and non-intrusive.

### Statistical analyses

We calculated the sample size required to detect an effect size of 0.6 (i.e. mean change in the outcome variable over time differs by at least 0.6 standard deviations between the two groups), at the α = 0.05 significance level, with 80% power to be 45 per group. An effect size of 0.6 is generally viewed as a medium to large effect. We sought to enrol 50 per group to allow for 10% withdrawal and non-compliance. These calculations were based on the changes found in an earlier study of *Jiangtang Xiaozhi* in people with diabetes [9].

Descriptive statistics and independent samples t-tests and 95% confidence intervals were used to compare the baseline characteristics of the two study groups (active treatment and placebo). All variables were visually inspected for normality. Fasting insulin, HOMA-IR, HOMA insulin sensitivity and HOMA beta cell were non-normally distributed. These data were log transformed to improve kurtosis and skewness before applying parametric statistical tests. HOMA estimates are usually not normally distributed [17]. Results were back transformed for presentation in the tables. The primary research objective was to compare change in glycaemic control after 16 weeks of treatment with either *Jiangtang Xiaozhi* capsules or placebo. Unless otherwise indicated, results are presented for the intention-to-treat (ITT) analysis. Analysis of covariance (ANCOVA) was used to model outcome at 16 weeks, by treatment group corrected for baseline level of the outcome variable. Each outcome variable was modelled separately. Baseline referred to data collected before any treatment was received. Post-hoc testing using pairwise comparisons of the estimated marginal means was used for within group analysis across the trial phases.

Statistical analyses were performed using SPSS for Windows version 17 (SPSS, Chicago, IL). A p value of <0.05 was considered statistically significant. Last observation carried forward (LOCF) was used for missing observations.

## Results

**Figure 1:**
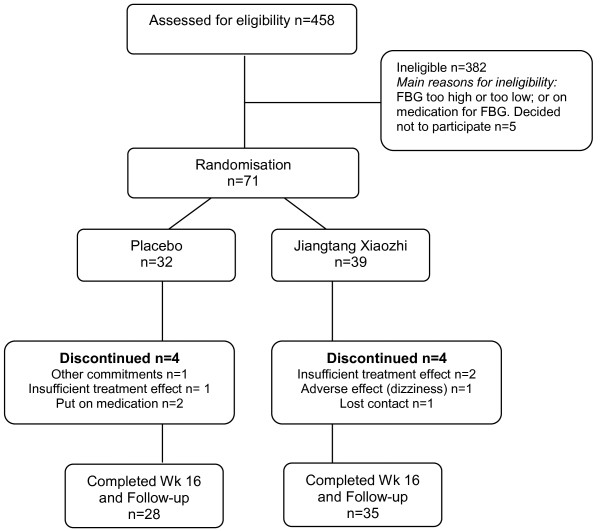
Participant flow through recruitment to trial completion.

A total of 458 subjects were assessed for eligibility and 71 subjects were enrolled, 39 were randomised to the intervention group and 32 to the placebo group. Eight randomly allocated participants did not complete the study; four in each group (see Figure 1).

Baseline characteristics are listed in Table 2. There was no significant difference between the *Jiangtang Xiaozhi* and placebo groups on age, sex, ethnicity, family history of diabetes, history of hypertension, smoking or use of cholesterol lowering or anti-hypertensive medication. Although the placebo group appeared to be heavier than the intervention group, there was no significant difference in BMI between the placebo (32.0 ± 8.0) and the CHM (29.8 ±4.9) groups (p =0.17). Measures of glycaemic control (FBG, two hr postprandial glucose and HbA1C) were similar across groups at baseline. Insulin resistance as calculated by HOMA-IR was not significantly different (p =0.37) between groups at baseline; mean levels were 2.12 ± 1.30 and 1.63 ± 0.91 in the placebo and CHM groups respectively. Forty-one patients were classified as having IGT and 30 with ‘controlled’ diabetes.

**Table 2:** Baseline demographic and clinical variables

**Variable**	**Placebo**	**JTXZ**
	**(*n *= 32)**	**(*n *= 39)**
*Age and Sex*		
Male/Female - *n*	18/14	15/24
Age - mean (range) age (years)	59.9 (40–75)	58.3 (36–83)
*Plasma glucose* - *mmol/L (SD)*		
Fasting	6.7 (1.0)	6.3 (*.*9)
Two hours after an oral glucose load	11.0 (2.9)	10*.*5 (2.3)
Glycosylated haemoglobin -% *(SD)*	6.4 (*.*7)	6.3 (*.*6)
Glycosylated haemoglobin - n ≥ 7%	6	5
Fasting insulin - *mmol/L (SD)*	15.5 (9.7)	11.8 (6.9)
*Serum lipids* - *mmol/L (SD)*		
Total cholesterol	4.5 (*.*9)	4.9 (1.0)
Triglycerides	1.7 (*.*9)	1.7 (1.3)
HDL cholesterol^	1.3 (*.*4)	1.5 (*.*4)
C-reactive protein - *mg/L (SD)*^	6.7 (5.3)	6.7 (7.6)
Systolic blood pressure - *mmHg (SD)^*	134.4 (14.0)	124.5 (13.3)
Diastolic blood pressure - *mmHg (SD)^*	81.7 (15.9)	76.4 (11.1)
Body mass index - *kg/m*^*2*^*(SD)*	32 (8.0)	29.8 (4.9)
Weight - *kg (SD)*	92.0 (28.5)	80*.*5 (15.1)
Waist - cm (SD)^a^	109.9 (22.1)	97.6 (11.1)
Waist-to-hip ratio - *waist/hips (SD)*	*.*97 (*.*19)	*.*88 (*.*07)
Diagnosed with type 2 diabetes - *n*	21	18
History of high lipids - *n*	18	23
Taking medication for cholesterol - *n*	14	15
Currently taking prescription medication - *n*	30	29
Currently taking vitamins, minerals or herbal supplements - *n*	24	24

### Fasting blood glucose

An analysis of covariance (ANCOVA), found that fasting blood glucose was not significantly different (p =0.73) at the completion of the treatment (Week 16) between the *Jiangtang Xiaozhi* group (6.3 ±1.1 mmol/L) and the placebo group (6.7 ± 1.3 mmol/L) (Table 3). No significant differences were detected between baseline and week 16 values within either the placebo or the *Jiangtang Xiaozhi* group for fasting blood glucose (placebo p =0.85; *Jiangtang Xiaozhi* p =0.85).

**Table 3:** Clinical measures

**Parameter**	***Jiangtang Xiaozhi***		**Placebo**		***P***
	**Baseline**	**Week 16**	**Baseline**	**Week 16**	
Fasting blood glucose (mmol/L)	6.3 ± 0.9	6.3 ± 1.1	6.7 ± 1.0	6.7 ± 1.3	0.70
Postprandial blood glucose (mmol/L)	10.45 ± 2.3	9.66 ± 2.6*	10.98 ± 2.9	10.60 ± 3.4	0.51
HbA1c (%)	6.4 ± 0.7	6.5 ± 0.7	6.3 ± 0.6	6.4 ± 0.6	0.62
Insulin (mmol/L)	11.8 ± 6.9	11.6 ± 5.5	15.5 ± 9.7	22.1 ± 25.9	0.04
HOMA-IR	1.63 ± 0.91	1.58 ± 0.74	2.06 ± 1.28	2.43 ± 1.59	0.06
HOMA%B	81.52 ± 34.5	83.5 ± 38.1	86.51 ± 36.78	98.83 ± 48.29	0.26
HOMA%S	81.84 ± 48.12	79.72 ± 44.14	72.78 ± 46.42	62.67 ± 38.48	0.34
Total cholesterol (mmol/L)	4.91 ± 1.03	4.96 ± 0.94	4.47 ± 0.85	4.56 ± 0.81	0.46
HDL cholesterol (mmol/L)	1.45 ± 0.44	1.54 ± 0.53	1.28 ± 0.30	1.24 ± 0.30	0.03
Triglycerides (mmol/L)	1.6 ± 0.64	1.6 ± 0.76	1.67 ± 0.93	1.59 ± 0.86	0.92
C-reactive protein (mg/L)	6.7 ± 7.6	4.66 ± 1.77	6.73 ± 5.25	5.18 ± 3.0	0.36
Weight (kg)	80.5 ± 15.1	80.4 ± 15.0	92.0 ± 28.5	91.8 ± 28.6	0.84
BMI (kg/m^2^)	29.8 ± 4.9	31.0 ± 1.8	32.0 ± 8.0	31.2 ± 1.8	0.64
Waist (cm)	97.6 ± 11.1	98.6 ± 12.4	109.9 ± 22.1	106.3 ± 18.6	0.05
Systolic blood pressure (mmHg)	124.5 ± 13.3	124.1 ± 15.5	134.4 ± 14.0	130.1 ± 16.1	0.96
Diastolic blood pressure (mmHg)	76.4 ± 11.1	71.7 ± 8.6	81.7 ± 74.3	74.3 ± 2.2	0.96

All data are means ± SD unless otherwise stated.

*within group statistically significant.

2 hr post prandial blood glucose levels were not significantly different between groups at the completion of treatment in week 16 (p =0.51) (Table 2). However, 2 hr postload glucose tended to decrease in the *Jiangtang Xiaozhi* group compared to baseline (p =0.03).

### Insulin

There was a significant difference between the placebo and the *Jiangtang Xiaozhi* group (p =0.04) at week 16. Mean levels of insulin at the end of the intervention, shown in Table 3, were 22.1 ± 25.9 mmol/L in the placebo group and 11.6 ± 5.5 mmol/L in the *Jiangtang Xiaozhi* group. At follow-up, the significant difference between the insulin levels of the two groups was no longer apparent.

Due to the high standard deviation at week 16 in the placebo group, data was examined for confounding factors such as outliers. None were identified. Recall that data were log transformed for ANCOVA and *t*-test analysis to address skewness and kurtosis and ensure the requirements of the statistical tests were met.

### Insulin resistance and sensitivity

After 16 weeks of treatment, there was a trend toward improved insulin resistance in the intervention group, although this narrowly failed to reach statistical significance (p = 0.06). HOMA-IR remained fairly steady in both groups from baseline until Week 16 when an increase in levels was observed in the placebo group. Levels in the placebo group had returned to baseline levels at follow up. From baseline to Week 24, there was no significant difference between the two groups (p = 1.0).

There was no significant difference between the two groups in HOMA%B or HOMA%S levels at Week 16.

Insulin sensitivity within the placebo group declined over the period of trial, although this failed to reach statistical significance (p =0.35). In the *Jiangtang Xiaozhi* group, insulin sensitivity improved moderately at the outset of the trial, remained stable and returned to similar pre-treatment levels at Week 24. The placebo group showed a slight decline in insulin sensitivity but returned to pre-treatment levels by Week 24. Mean levels were 70.3 ± 39.2 and 74.4 ± 41.1 in the placebo and *Jiangtang Xiaozhi* groups respectively.

### Cholesterol

For HDL cholesterol, there was a significant improvement in the *Jiangtang Xiaozhi* group compared to placebo at the end of the treatment period (p = 0.03). Mean levels in the placebo group had slightly decreased (1.24 ± 0.30 mmol/L) compared to baseline and increased in the *Jiangtang Xiaozhi* group (1.54 ± 0.53 mmol/L). At follow up, there was no longer a significant difference between the two groups at follow up at week 24 (p = 0.11).

There was no significant difference in total cholesterol (p = 0.46) or triglycerides (p = 0.92) between the treatment and placebo group at the completion of the treatment phase of the trial.

### Quality of life

We found no post-treatment in any of the eight dimensions of the SF-36 between the *Jiangtang Xiaozhi* group and the placebo group. It is of interest, however, that our clinical trial cohort had poorer quality of life on three of the eight dimensions of the SF-36: vitality, role limitations due to emotional problems and mental health when compared with the age relevant cohort (55–64 yrs) and all-age Australian norms [18].

### Other measures

At 16 weeks, there was no significant difference between the two groups on BMI, waist circumference, CRP, blood pressure or HRQoL. No differences were found at follow up eight weeks later.

Behaviour change in physical and dietary habits was measured at different time intervals throughout the trial as it is known to affect blood glucose and insulin levels. There was no significant change in any group on nutritional intake or physical activity from baseline to the completion of the trial.

### Safety and adverse events

The liver function of all participants was assessed at the baseline and throughout the trial to monitor for any possible adverse reactions. *Jiangtang Xiaozhi* was well tolerated with no serious adverse events. There were no significant abnormalities in liver function noted in either group.

No fatalities or major adverse events occurred during the trial. One participant in the intervention group developed moderate dizziness within 24 hours of the medication. The participant stopped the medication for 24 hours and the dizziness ceased. A rechallenge produced similar symptoms and as a result the participant was withdrawn from the trial. This patient was later found to be in the *Jiangtang Xiaozhi* group.

Blinding was effective with only 25% of participants correctly identifying their group in the first four weeks and 27% in the final four weeks of the intervention.

## Discussions and conclusions

### Effect on blood glucose

In the present study, we found no significant differences on any of the glycaemic outcome measures between the *Jiangtang Xiaozhi* and placebo groups at completion of the treatment. However, the study yielded three positive findings on secondary outcomes. First, levels of insulin resistance (HOMA-IR) were lower than those in the placebo group. Second, serum insulin slightly decreased in the treatment group compared to worsening levels in the placebo group, resulting in a borderline significant difference between groups. Third, HDL cholesterol was significantly improved in *Jiangtang Xiaozhi* group compared to the placebo. The absence of a detectable glycaemic measures in this trial contradicts with the previous clinical trial. In this earlier trial in a group with type 2 diabetes, plasma glucose levels reduced significantly from baseline after 8 weeks treatment by −1.71 ± 2.52 mmol/L compared to −0.72 ± 4.17 mmol/L in the pioglitazone group [9]. Postprandial plasma glucose and HbA1c also both showed a significant difference compared to baseline (11.41 ± 2.63 mmol/L to 9.91 ± 1.93 mmol/L and 7.35 ± 1.87% to 6.73 ± 1.02%, respectively). The reasons for this discrepancy can be complex. However, possible explanations may include: (a) this herbal medicine may be ineffective in treating elevated glucose levels in people with IGT; (b) it may be that the size of the sample was not sufficiently large to detect an effect, particularly given the transient nature of IGT; (c) an effect might only occur with greater symptom severity at baseline [19]. The fourth possibility is that the dosage of *Jiangtang Xiaozhi* was not adequate. Our clinical trial cohort was largely overweight and obese with a mean BMI of 30.8 kg/m^2^, which may also affect the efficacy of an intervention [20].

A significant improvement within the *Jiangtang Xiaozhi* group on postprandial plasma glucose levels at the completion of the treatment was identified compared to baseline. Measuring change from baseline is an acceptable and meaningful statistic where baseline levels are comparable between the intervention and placebo groups, which in our case they were. Hyperglycaemia in prediabetes is primarily postprandial in nature. The body is unable to control blood glucose adequately after a loading of ‘sugar’. It is these postprandial ‘spikes’ in blood glucose levels that are thought to be toxic to the beta-cells and cause them to dysfunction [21]. Perhaps *Jiangtang Xiaozhi* may be of assistance in reducing these ‘spikes’.

### Effect on insulin

Insulin resistance is a decreased responsiveness of target tissues - skeletal and myocardial myocytes, hepatocytes, and adipocytes - to normal levels of circulating insulin (Setsi 2006). In our placebo group, higher levels of insulin resistance (HOMA-IR) accompanied higher levels of serum insulin. This is to be expected. Greater serum insulin levels are seen in those with higher insulin resistance. A smaller insulin response is anticipated in those with better insulin sensitivity. At week 16 of treatment, there was a trend for insulin resistance to improve in the *Jiangtang Xiaozhi* group compared to the placebo group, but the change narrowly missed statistical significance.

However, the level of change detected in insulin resistance in our trial may only have marginal clinical significance. Cut-off values for normal HOMA-IR are considered to lie somewhere between 2.5 and 4.1 [17,22,23]. At these values our clinical trial cohort would be considered to be in the non-clinical range at the start of the trial with levels of 2.12 ± 1.30 and 1.60 ± 0.92 in the placebo and *Jiangtang Xiaozhi* groups respectively. The 3 month clinical trial of the herbal extract, berberine, found a clinical and statistically significant difference with HOMA-IR reducing from 3.9 to 2.44 in people with diabetes not IGT [24]. Perhaps our clinical trial cohort was overall too well at baseline and our sample did not allow for sufficient power to detect change from these baselines.

In the present study, mean beta-cell function (HOMA%B) increased in the placebo group from 87% to 99% but not to a statistically significant degree while remaining stable in the *Jiangtang Xiaozhi* group (82% to 84%). These results could be interpreted as a trend towards improvement in the beta-cell function of the placebo group. However, beta-cell function needs to be interpreted in the context of serum insulin, insulin sensitivity and insulin resistance.

Typically a beta-cell or HOMA%B value that is closer to 100% is associated with better beta-cell function [17,25-27]. Why then in the placebo group, compared to the *Jiangtang Xiaozhi* group, would insulin secretion increase, insulin sensitivity decrease but beta-cell function (HOMA%B) appear to improve? One explanation may be that a ‘high’ HOMA%B does not always equate to better beta-cell functioning but perhaps the opposite.

When insulin sensitivity is improved, beta-cell activity may be reduced – the beta-cells of the pancreas simply don’t have to work as hard anymore [17]. This explanation is supported by several longitudinal studies which have shown that decreased beta-cell function as represented by HOMA%B does not, on its own, seem to predict the development of diabetes. A five year study of 12,924 non-diabetic Koreans examined the role of HOMA%B in predicting the development of diabetes. They found that the HOMA%B baseline value was actually higher in those who went on to develop diabetes [28]. Another study which utilised HOMA to predict the development of diabetes concluded that whereas low insulin secretion may be adequate for an insulin sensitive patient, the same level of beta-cell function may be inadequate for another patient [29]. The developers of the HOMA instrument have indeed pointed out that “HOMA-%B is a measure of beta-cell activity, not of beta-cell health or pathology” and that HOMA%B values need to be considered alongside HOMA%S and HOMA-IR [17]. Therefore what may have been happening in the placebo group was the natural progression of diabetes: an increase in insulin secretion combined with a rise in beta-cell activity (HOMA%B) coupled with a rise in insulin resistance (HOMA-IR) indicating that the beta-cells are working harder. Whereas in the *Jiangtang Xiaozhi* group the insulin measures, stable serum insulin, stable insulin sensitivity and reduced insulin resistance compared to the worsening insulin measure in the placebo group indicate that progression has perhaps stalled but not reversed.

Nonetheless, the degree to which the intervention appeared to maintain insulin sensitivity levels, restrain insulin secretion and thereby help preserve beta-cell function does warrant further investigation. Worsening of impaired glucose tolerance, progressing to frank diabetes is generally accepted as a consequence of insulin resistance, impaired insulin secretion and pancreatic beta-cell failure [30-32]. While the relative contribution of each of these factors is still a subject for debate, we do know that insulin resistance plays a key role and this is evidenced by a number of longitudinal and cross-sectional studies.

### Effect on cholesterol

People with diabetes often have abnormally low levels of HDL cholesterol and high levels of triglycerides [33]. There is also a strong association between dyslipidaemia and insulin resistance [34]. We found that high-density lipoprotein cholesterol (HDL), otherwise known as the ‘good’ cholesterol, improved post-treatment in the *Jiangtang Xiaozhi* group compared to the placebo group. Cholesterol lowering medication was being taken by nearly all our clinical trial participants. When analysed as a covariant there was no significant effect exerted by cholesterol lowering medication consumption on any of the lipid results.

The mean post-treatment increase of 0.10 mmol/L in HDL-cholesterol in the *Jiangtang Xiaozhi* group represents a 6% change from baseline and is thus of some clinical significance. In a pooled analysis of four clinical trials of statins, individuals with a ≥7.5% increase in HDL cholesterol in conjunction with lowered LDL had a reduced incidence of coronary atherosclerosis [35].

A new approach toward treating dyslipidaemia alongside high blood glucose levels has been to target insulin resistance [36]. Thus, a possible explanation for the improved HDL levels in the *Jiangtang Xiaozhi* group may have been improved insulin resistance. Our results indicate *Jiangtang Xiaozhi* both improves HDL levels and stabilises insulin. This is a particularly encouraging clinically relevant finding as it signifies the potential of the *Jiangtang Xiaozhi* to treat two conditions and thus avoid some of the problems inherent with polypharmacy.

### Limitations of our study

Our study has several limitations. Firstly, our sample size may have lacked sufficient statistical power to detect a clinically significant change in FBG and to adequately account for the transient nature of people with IGT [19]. Secondly, the intervention period may have been too short to allow for the natural progression of impaired glucose tolerance. A further limitation relates to the methodology of the outcome measures. We used HOMA to assess insulin resistance and sensitivity as a cost effective method with validity for clinical trials. The use of the euglycaemic clamp method to assess insulin sensitivity may have provided a more accurate result. Clamp methods are not feasible in large studies. Likewise we used only one insulin measure and this may not have sufficiently accounted for intra-individual variation. A final limitation was dosage. The dosage used in our trial was significantly less than that used in the trial of *Jiangtang Xiaozhi* in people with diabetes (34 grams per day compared to 75 grams per day in the first study). It is also likely that therapeutic doses weren’t reached by some participants. The regime of three tablets three times a day is a difficult dosage regime. Although a final pill count was undertaken not all participants returned leftover medication. Simpler, less frequent dosing regimens result in better compliance [37].

In summary, although *Jiangtang Xiaozhi* did not significantly change blood glucose levels, the intervention was associated with some positive effect on insulin and HDL. The positive results of *Jiangtang Xiaozhi* in reducing postprandial glucose indicate that a higher level of baseline severity in blood glucose symptoms might yield more reliable findings. Our analysis was considerably underpowered. A longer study, in line with other interventions in this population group, to allow for the natural progression of the disease may also bring forth an effect on fasting blood glucose. The strength of this study was that it was a robust double-blinded, placebo controlled trial conducted according to rigorous scientific methodology.

The safety of this herbal formulation and its components has been demonstrated in animal and human studies. The lack of, or minimal, side effects provide a considerable advantage over many of the current pharmaceutical treatments used for the treatment of prediabetes and controlled diabetes.

In light of the growing epidemic of diabetes worldwide, preventing or delaying the onset of diabetes may likewise reduce the microvascular and macrovascular complications of the disease. It is worthwhile investigating the potential of *Jiangtang Xiaozhi* to decrease blood glucose levels and reduce or prevent the incidence of diabetes in a longer, adequately powered trial.

## Competing interests

The authors declare that they have no competing interests.

## Authors’ contributions

SG researched data, conducted clinical trial, wrote manuscript. DC contributed to clinical trial design and discussion, reviewed/edited manuscript. VW contributed to clinical trial design and discussion, reviewed/edited manuscript. JL contributed to trial design, reviewed/edited manuscript. AB contributed to discussion, reviewed/edited manuscript. HK contributed discussion, reviewed/edited manuscript. All authors read and approved the final manuscript.

## Pre-publication history

The pre-publication history for this paper can be accessed here:

http://www.biomedcentral.com/1472-6882/13/104/prepub
